# Retinal blood flow reduction after panretinal photocoagulation in Type 2 diabetes mellitus: Doppler optical coherence tomography flowmeter pilot study

**DOI:** 10.1371/journal.pone.0207288

**Published:** 2018-11-08

**Authors:** Youngseok Song, Tomofumi Tani, Tsuneaki Omae, Akihiro Ishibazawa, Takafumi Yoshioka, Kengo Takahashi, Masahiro Akiba, Akitoshi Yoshida

**Affiliations:** 1 Department of Ophthalmology, Asahikawa Medical University, Asahikawa, Japan; 2 R&D Division, Topcon Corporation, Tokyo, Japan; Boston Medical Center, Boston University School of Medicine, UNITED STATES

## Abstract

To use a Doppler optical coherence tomography (DOCT) flowmeter to investigate segmental retinal blood flow (RBF) and sum of the segmental RBFs (SRBF) changes after panretinal photocoagulation (PRP) was used to treat type 2 diabetes mellitus with severe diabetic retinopathy (DR). Data from five patients with proliferative DR (PDR) (mean age 51.9 ± 10.5 years) was analyzed. The vessel diameter (D), average velocity (V), and retinal blood flow (RBF) in veins were measured using a DOCT flowmeter before and four weeks after PRP. Segmental RBF from inferotemporal (IT), superotemporal (ST), inferonasal (IN), and superonasal (SN) veins were measured, and SRBF was defined as the sum of these measurements. All data were analyzed by Wilcoxson test. After PRP, there were statistically significant decreases in the every segmental D, V, RBF (*P<0*.*03*) and SRBF (*P = 0*.*002*). The other parameters showed no statistically significant differences (*P*>0.05). The DOCT flowmeter has the potential to be a clinically useful tool to noninvasively evaluate the changes in retinal circulation during PRP in patients with PDR.

## Introduction

Diabetic retinopathy (DR), a leading cause of blindness in industrialized countries, can be effectively treated using panretinal photocoagulation (PRP), which induces regression of retinal neovascularization (NV) in patients with proliferative diabetic retinopathy (PDR).[1–4] Though the effect of PRP is generally evaluated by assessing reduction from leakage from NV using fluorescein angiography (FA) in clinical practice, FA can have serious complications due to the intravenous injection of fluorescein dye. The incidence rate of a severe reaction was 1:1900, and that of death was 1:222,000.[5] Therefore, developing non-invasive methods to evaluate the effect of PRP is of paramount importance.

Several clinical studies in patients with PDR showed reduction of regional retinal blood flow (RBF) and total RBF (TRBF) after PRP using laser Doppler velocimetry (LDV) and photographic measurements of vessel calibers.[6]^,^[7, 8] However, the segmental RBF and TRBF after PRP have not yet been reported in a single study, most likely because acquiring successful RBF measurements with the LDV system requires a highly skilled operator and a substantial amount of time.

To replace the LDV system, different techniques have recently been developed based on Doppler optical coherence tomography (DOCT). A study based on the DOCT system showed that there was no significant reduction of TRBF after PRP in patients with poorly controlled type 2 diabetic mellitus (DM).[9] However, the technique used in this study (double circle-based calculation method) measured RBF at only a few moments (< one cardiac cycle), despite the fact that RBF measurements must be measured continuously for at least one cardiac cycle in order to obtain accurate blood velocity in the pulsatile retinal vessels.[9]

To overcome these disadvantages, we recently developed a commercially available semi-automated DOCT instrument (Topcon Corp., Tokyo, Japan) with a novel software featuring a segmental scanning method which enables measurements over one cardiac cycle. We confirmed that the DOCT flowmeter enabled accurate and physician-friendly measurement of segmental and sum of the segmental RBFs (SRBF) without any specific skills.[10, 11] The goal of this study was to investigate the changes in segmental RBF and SRBF during PRP in poorly controlled PDR patients using the DOCT flowmeter.

## Methods

### Design

All methods and experimental protocols were carried out in accordance with the guidelines approved by the institutional review board of the Asahikawa Medical University (Asahikawa Medical University Independent Ethics Committee: AMUIEC) and the tenets of the Declaration of Helsinki. The specific protocols of the study were approved by AMUIEC (the approved number of the study was 17114). All subjects provided written informed consent to participate in this research after receiving a complete explanation of the study design and protocol. 10 patients with DR (male : female = 4 : 6 ) were recruited for the study at Asahikawa Medical University, Asahikawa, Japan. All subjects underwent an ophthalmologic examination, including review of medical history, slit-lamp biomicroscopy, intraocular pressure (IOP) measurement, and funduscopic examination using a 90-diopter lens. The mean arterial blood pressure (MABP) was calculated by the formula ([2 × diastolic BP] + systolic BP)/3. The ocular perfusion pressure (OPP) was calculated by the formula 2/3MABP—IOP.

### Inclusion criteria and diagnosis of PDR

Using the Early Treatment of Diabetic Retinopathy Study criteria, cases with type 2 DM including an ETDR score of more than level 61 that were classified as having PDR without any history of prior PRP and/or a history of eye surgery were recruited.[12, 13] Cases with vitreous hemorrhage during the course of the study were excluded to ensure that the scans were successfully obtained. The baseline RBF measurements were taken immediately before PRP. A certified ophthalmic specialist (TO) diagnosed DR in all patients using FA and fundus photographs, and another specialist (AI) applied PRP. These clinicians were masked to the DOCT flowmeter results.

### DOCT flowmeter

The retinal vein blood circulation was measured using the DOCT flowmeter.[10] As described in detaile previously, our system is based on a commercially available spectral-domain OCT system (3D OCT-2000 FA, Topcon Corp.) operated at an 800-nm wavelength range.[10] The image-capturing software was modified for Doppler imaging, and the image-processing software was newly developed to measure the RBF according to a previously described segmental scanning method.[10] A total of 180 datasets were captured for approximately 2 seconds with a scan length of 1mm. Multiple vessels were automatically detected, and only veins with a diameter of more than 80 μm were measured.[10] It is generally accepted that the measurement accuracy of velocity is decreased when the Doppler angle is close to 90 degrees, i.e., when the optical axis and flow vector are set to nearly perpendicular.[10] In such cases, the amount of Doppler shift or phase difference becomes equal to 0.[14] The measured flow rates in the arteries and veins seemed to be stable under 85 degrees, however, it was overestimated when the Doppler angle exceeded 86 degrees.[10] Therefore, we excluded the measurement results when the Doppler angle was calculated to be more than 86 degrees.

### Imaging procedure and analysis

**Fig 1:**
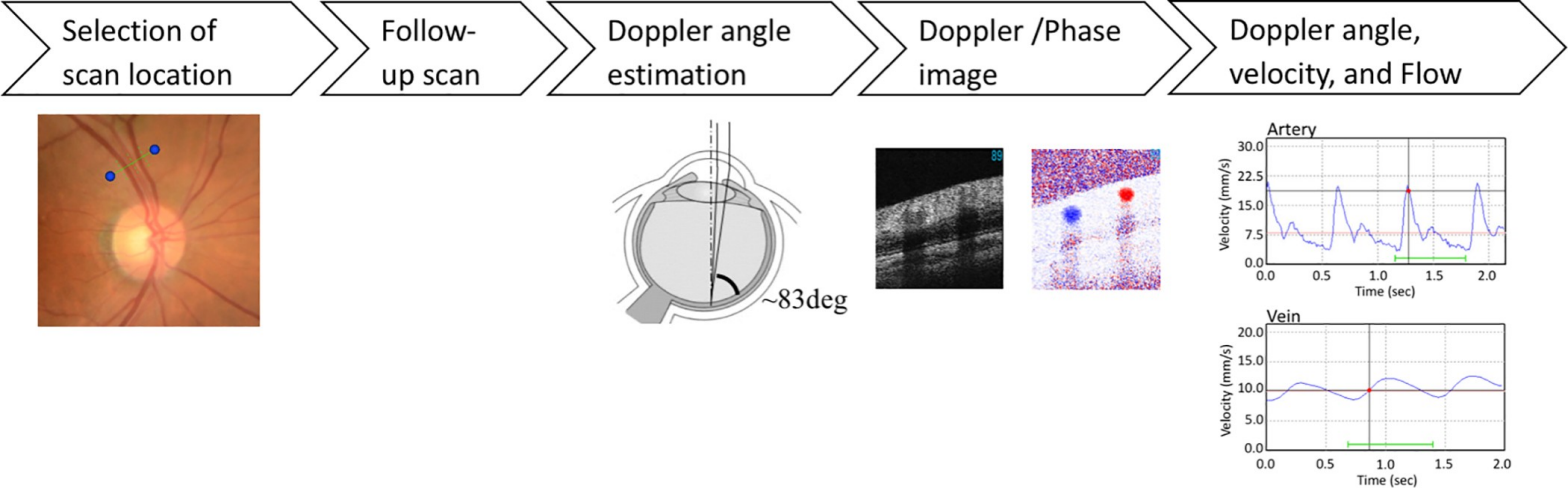
Image procedure and analysis of measuring retinal blood flow using Doppler optical coherence tomography flow meter (DOCT flowmeter).

Retinal blood flow was measured according to a previously described method.[10, 11] As shown in Fig 1, Before BF imaging, a single color fundus image centered at optic disc was captured then region of interest of retinal vessel which is typically one disc diameter away from center of the optic disc was manually chosen. Scan location was also set in this procedure to make sure that it is almost perpendicular to the blood vessel. Flow velocity was calculated using the Doppler angle which is the angle between the incident bean and flow direction, and the amount of Doppler shift (phase image). To calculate the Doppler angle, a pair of scans was implemented before main BF measurement. Detailed description was found in previous literature.[10, 11] In brief, two scans were parallel each other but perpendicular to the blood vessel with Y = 100um apart. By using the phase image, Doppler angle was calculated by identifying the vessel center. For a small segment, the blood vessel can be treated as a straight line. Then, the measurement of cardiac cycle is performed for ~2 seconds. During this scan, scan location is adjusted accordingly by using retinal motion tracking function. During this ~2 seconds measurement, one can see at least one cardiac cycle pulsation curve for most of the cases. Doppler shift image (phase image) was calculated by taking the phase difference between adjacent scans as described previously.[10, 11] Collected phase images were registered and summed to find the vessel location, its center, and diameter by automated software. Flow is then calculated using vessel diameter and velocity.

A single color fundus image centered at optic disc was captured then region of interest of retinal vessel which is typically one disc diameter away from center of the optic disc was manually chosen. Scan location was set in this procedure to make sure that it is almost perpendicular to the blood vessel. Flow velocity was calculated using the Doppler angle which is the angle between the incident beam and flow direction, and the amount of Doppler shift (phase image). A pair of scans was implemented before main BF measurement to calculate the Doppler angle. Two scans were parallel each other but perpendicular to the blood vessel with Y = 100um apart. By using the phase image, Doppler angle was calculated by identifying the vessel center. The measurement of cardiac cycle is performed for ~2 seconds. During this ~2 seconds measurement, one can see at least one cardiac cycle pulsation curve. Doppler shift image (phase image) was calculated by taking the phase difference between adjacent scans. Collected phase images were registered and summed to find the vessel location, its center, and diameter by automated software. Flow is then calculated using vessel diameter and velocity.

### PRP procedure

We began to apply PRP approximately two to three disc diameters away from the center of the macula and peripherally to the equator, i.e., 1,000 to 1,200 spots, 0.1 to 0.2 seconds, and 200- to 400-micron argon laser burns at four sites in the following order: inferiorly, temporally, nasally, and superiorly. PRP was applied during multiple sessions 2 weeks apart (about 400 spots/session) starting from the inferior fundus location. The DOCT flowmeter measurements were performed 4 weeks after the last treatment session. Fluorescein fundus angiography was also performed 1 month following PRP.

### Study protocol

**Fig 2:**
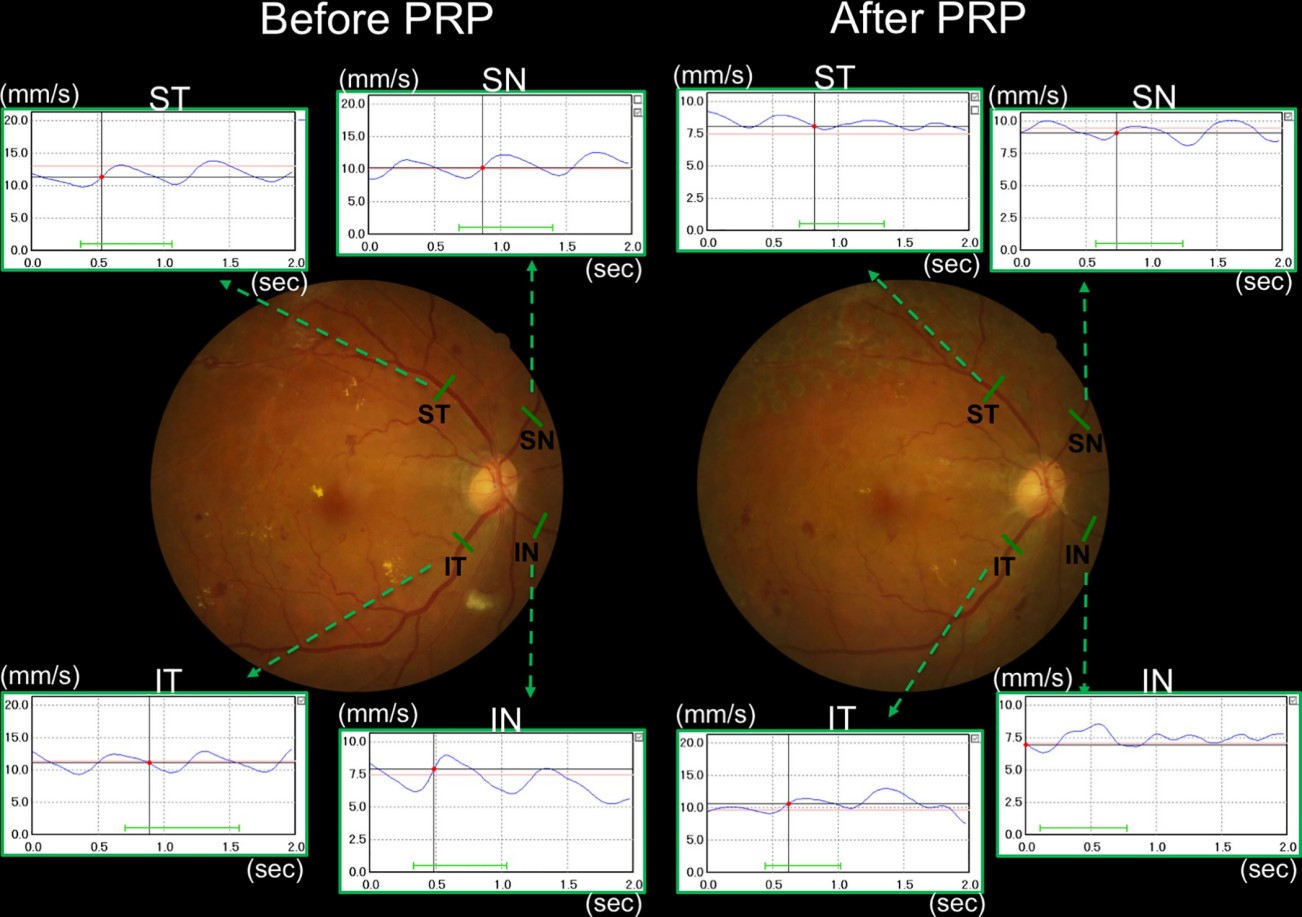
Examples of the site of venous measurements before (baseline) and after panretinal photocoagulation (Post PRP) and the waveforms of the blood velocity for the veins in before and after PRP eyes. The green bars indicate the inferotemporal (IT) vein, superotemporal (ST) vein, inferonasal (IN) vein, and superonasal (SN) vein. Each waveform of the veins were acquired by Doppler OCT flowmeter. The vertical lines indicate the retinal blood velocity (mm/sec) and the horizontal lines indicate time (sec). Before PRP, before starting PRP; After PRP, four weeks after completing PRP.

An examiner (TT) performed all measurements. After the pupils were dilated with a 0.5% tropicamide eye drop (Santen Pharmaceutical Co., Osaka, Japan), the subjects were required to rest for at least 5 minutes while sitting in a quiet, dimly lit room at a temperature of 25˚C, after which their BP was measured in the left arm. The DOCT flowmeter was assessed after the BP and IOP measurements. The subjects abstained from coffee for at least 24 hours before the measurements. Considering the measuring limit of vessel diameter using DOCT flowmeter (>50μm), the relatively large veins (diameter of 80 μm or more) chosen for measurement had relatively straight segments one disc diameter away from the optic disc. When there were two or more veins in a sector, the biggest one was chosen for the measurement because smaller veins were more likely to give unsuccessful measurements because veins get smaller after PRP. We measured the D, V, and RBF of the inferotemporal (IT), superotemporal (ST), inferonasal (IN), and superonasal (SN) veins (Fig 2), and we defined the SRBF as the sum of all measured veins (IT + ST + IN + SN), and vessel D as the automatically calculated flow image of the horizontal lumen. The waveforms of the veins in the case were shown in Fig 2.

### Statistical analysis

The data were analyzing using Prism 6.0 (GraphPad Software, Inc.). All data were expressed as the mean ± standard deviation. The data were analyzed using Wilcoxon test to compare the measurements before and after PRP. *P*<0.05 was considered statistically significant.

## Results

The demographics of the subjects are shown in Table 1. The mean age of the five subjects was 51.9 ± 10.5 years. The average time from the onset of DM was 11.0 ± 2.9 years (range, 8–16 years). The average hemoglobin A1c (HbA1c) was 9.3 ± 2.0%. All subjects had systemic hypertension and type 2 diabetes. Before PRP therapy, seven subjects (70%) out of 10 had severe non-perfusion area (NPA), two (20%) had pure neovascularization elsewhere (NVE) confirmed by FA (Subjects 1 and 3), one (10%) had both NVE and neovascularization of the disc (NVD) (Subject 2), and none had neovascularization of the iris (NVI). The average number of laser spots applied was 1,217.6 ± 77.0. 8 weeks after PRP the HbA1c, IOP, systemic BP, diastolic BP, MABP, OPP, and heart rate values were similar to before PRP (*P* = 0.36, 0.36, 0.31, 0.89, 0.39, 0.57, and 0.14, respectively).

**Table 1:** Patient demographics before panretinal photocoagulation (at baseline).

Subject	Age	Gender	Duration	Log MAR	HbA1c	HT	IOP	sBP	dBP	Mean BP	OPP	PRP Spots
1	45	M	10	-0.08	9	Yes	14	141	68	92	48	1142
2	50	F	15	-0.08	11.2	Yes	14	137	73	94	49	1241
3	34	M	9	0.15	12.7	Yes	21	154	87	109	52	1362
4	67	M	12	0	6.8	Yes	18	145	98	114	58	1132
5	45	M	8	0	10.9	Yes	16	148	77	104	53	1256
6	56	F	13	0.1	6.8	Yes	16	139	72	94	47	1211
7	49	F	8	0	10.9	Yes	15	151	80	104	54	1131
8	67	F	11	0	8.5	Yes	14	144	71	95	50	1156
9	46	F	8	0.1	7.8	Yes	17	138	75	96	47	1278
10	60.0	F	16	-0.3	8.5	Yes	19	149	81	104	50	1267

Data are expressed as the mean ± standard deviation (SD) of the mean. DM, diabetes mellitus; Duration, duration of DM (years); logMAR, logarithm of the minimum angle of resolution visual acuity; HbA1c, hemoglobin A1c (%); IOP, intraocular pressure; sBP, systolic blood pressure (mmHg); dBP, diastolic blood pressure (mmHg); Mean BP, mean blood pressure (mmHg); HT, hypertension; OPP, ocular perfusion pressure (mmHg); HR, heart rate (bpm); PRP, panretinal photocoagulation; PRP spots, total number of PRP spots.

**Fig 3:**
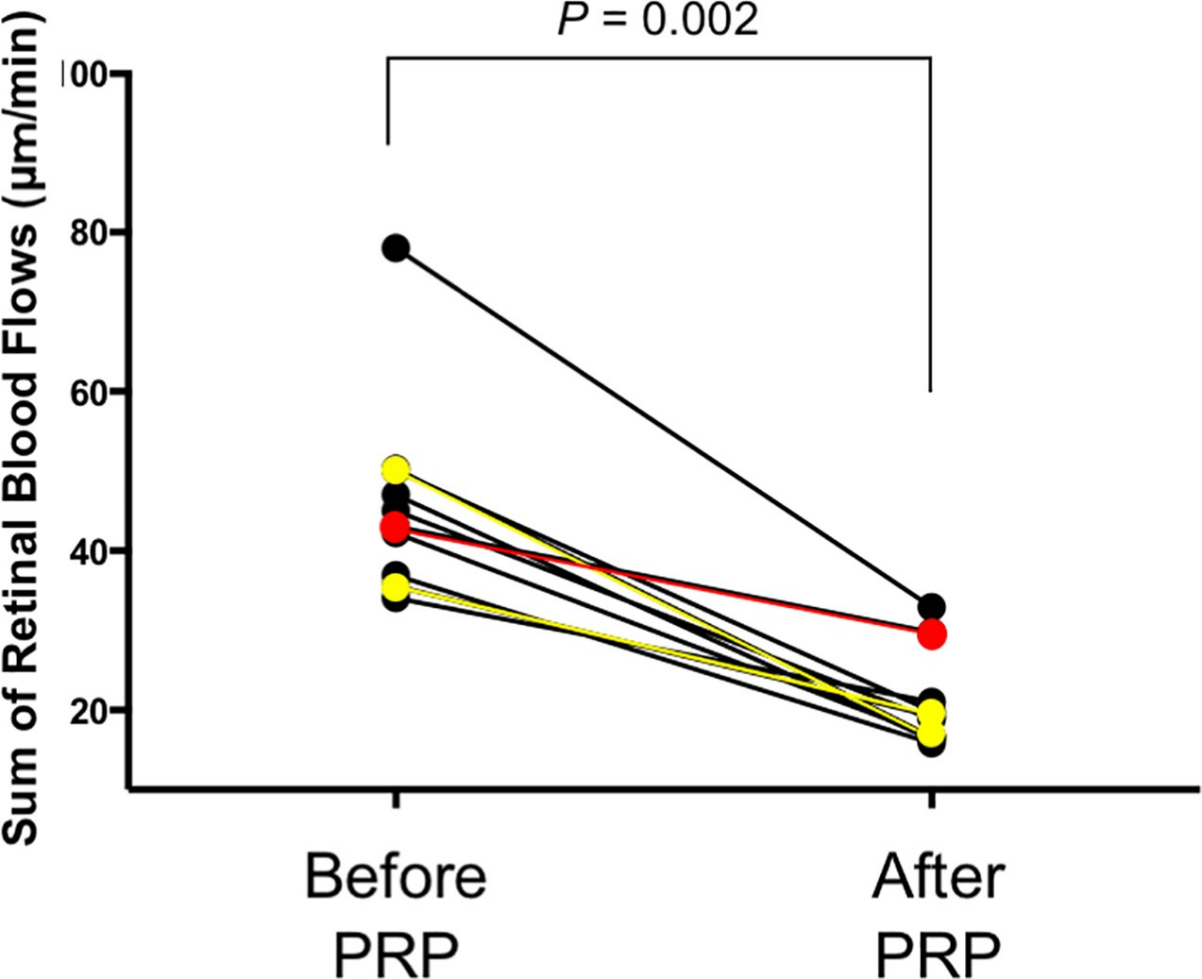
Sum of the retinal blood flows (SRBF) before and after panretinal photocoagulation (PRP). SRBF decreased significantly after PRP (*P = 0*.*002*, Wilcoxon test). In FA after PRP, though two subjects with NVE and/or NVD (Subjects 1 and 2: yellow lines) had regression of NV, one subject (Subject 3: red line) with NVE did not have regression of NVE. No subject had the formation of new NV. Before PRP, SRBF before starting PRP; After PRP, SRBF four weeks after completing PRP.

The average D, V, and RBF of all segmental vessels are shown in Table 2. The D, V and RBF of all segments decreased significantly after PRP (*P* < 0.03). SRBF in all subjects also decreased significantly (*P* = 0.002) (Fig 3).

**Table 2:** Flow parameter changes before and after panretinal photocoagulation in all segmental vessels.

	Diameter, μm		Velocity, mm/s		RBF, μL/min	
Segmental Site	Before PRP	After PRP	Before PRP	After PRP	Before PRP	After PRP
IT	164.6 ± 22.1	139.2 ± 17.1*	12.2 ± 2.8	7.7 ± 2.0*	14.7 ± 3.9	6.6 ± 1.8*
ST	161.6 ± 19.3	129.0 ± 23.8*	11.8 ± 4.4	7.3 ± 4.0*	16.0 ± 11.1	6.8 ± 5.4*
IN	131.1 ± 19.6	107.0 ± 17.1*	11.5 ± 3.2	6.1 ± 2.7*	18.5 ± 3.3	3.7 ± 1.6*
SN	112.0 ± 12.7	100.3 ± 10.8*	11.2 ± 3.8	6.5 ± 3.3*	17.0 ± 3.8	3.6 ± 2.5*

Data are expressed as the mean ± standard deviation (SD) of the mean.

*P< 0.*05* was considered statistically significant in the comparisons of measurements before and after PRP using Wilcoxon test. PRP, panretinal photocoagulation; RBF, retinal blood flow; IT, inferotemporal vein; ST, superotemporal vein; IN, inferonasal vein; SN, superonasal vein. NVE; neovascularization elsewhere, NVD; neovascularization of the disc, NV; neovascularization.

## Discussion

In the current study, we evaluated the effect of PRP in both the segmental RBF and SRBF using the semi-automated DOCT flowmeter that we developed. The current findings showed that the segmental RBF and TRBF significantly decreased after PRP (Table 2 and Fig 3). These findings are consistent with the results of previous clinical reports using a laser Doppler technique.[6–8] To the best of our knowledge the current report shows the changes in all segmental RBF (IT, ST, IN, and SN veins) and SRBF after PRP in a single report for the first time.

Though an advantage of the laser Doppler method is the ability to measure absolute blood velocity and RBF, measuring RBF with this technique requires a skilled operator and takes a long time. To overcome these issues, several flow meters equipped with the various DOCT methods have been developed.[14, 15] Using our DOCT flowmeter, we not only successfully measured segmental RBF quickly, but also obtained SRBF in patients with poorly controlled DM who needed PRP.

Doppler angle detection is an important factor for measuring RBF with the DOCT technique. However, the bidirectional two-beam technique is a reliable method that does not require measurement of the Doppler angle.[14] Although the two-beam technique is suitable for Doppler angle detection if the vessel is nearly parallel to the fundus, the technique requires complex optical settings that use two-beam and two-spectrometer configurations with polarization optics. In contrast, our single-beam–based segmental-scanning DOCT[10] was incorporated into a commercially available spectral-domain OCT system with modified software.[10] In addition, the technical strength of our DOCT flowmeter was the use of fully automated grading and calculation techniques for the RBF measurements in the retinal vessels. One of the reasons why the RBF decreased after PRP could be the alteration of oxygen tension in the retina. Local increases in retinal oxygen tension are thought to occur as the result of laser photocoagulation because PRP destroys the high oxygen-consuming photoreceptors in the outer retina, and PRP induces regression of retinal neovascularization in patients with severe NPA and PDR.[16–18] Elevated oxygen tension in the inner retina induces a retinal vasoconstrictive response.[19–21] Therefore, the current RBF reduction is a reasonable response for both vasoconstrictive reactions, possibly due to the increased retinal tissue oxygen tension following the reaction of the decreased metabolic demand of the retinal tissue after PRP.

In the study, there were three subjects with NV (Subjects 1, 2 and 3). The two subjects (subject 1 and 3) out of three had regression of NV after PRP and their SRBF reduced more than 45% after PRP. On the other hand, one subject (subject 2) with active NV even after PRP had only 30% of reduction in SRBF possibly due to the less effective PRP, suggesting that DOCT flowmeter might be able to assess the efficacy of PRP with the rate of RBF reduction after PRP. However, the population of the current study is relatively small and there were only three subjects with NV in the study. Further studies with larger cohort of PDR with NV would confirm the hypothesis.

The RBF has an autoregulation system in which the tissue can maintain relatively constant blood flow despite some variations in the OPP,[22] and it is known that autoregulation of the RBF might be impaired in type 1 DM.[23] Though it is possible that decreased OPP may result in decreased RBF, we confirmed that there was no significant change (*P* = 0.68) in OPP between before and after PRP (Table 1).

The current study has some limitations. First, we had a relatively small number of patients in this study. Though the current study revealed useful information that is helpful in understanding the changes in RBF after PRP in patients with DR, further studies with a larger cohort are warranted. Second, we performed follow-up RBF measurements after PRP, however, the current study did not clarify the direct relationship between RBF reduction and the improvement of DR, such as regression of NV on FA images. Further studies are needed to assess the direct relationship between RBF reduction and the improvements of DR after PRP. Third, although we measured HbA1c we did not measure the serum glucose levels before and after PRP, which affected the changes in RBF and endothelial dysfunction in our in vivo study using LDV.[24] Therefore, the current study did not exclude the potential effects of serum glucose level on RBF changes. In addition, there were several veins which were not measured in the study because DOCT flowmeter has the limitation of vessel size to measure, i.e., DOCT flowmeter can measure vessels with more than 50 μm. Improving the resolution of measurements using DOCT flowmeter is expected.

In conclusion, our DOCT flowmeter showed that the segmental RBFs and the SRBF decreased 4 weeks after PRP in PDR subjects with poorly controlled type 2 DM. The DOCT flowmeter might be a useful method to noninvasively evaluate the effects of PRP in patients with PDR.
